# A single-blind, parallel trial of l-hydroxyproline in healthy adult subjects

**DOI:** 10.1007/s00240-015-0765-5

**Published:** 2015-04-01

**Authors:** Saori Akiduki, Haruo Ito, Koji Morishita, Ayako Kamimura

**Affiliations:** 1 Healthcare Products Development Center, KYOWA HAKKO BIO CO., LTD., 2, Miyukigaoka, Tsukuba, Ibaraki 305-0841 Japan; 2 Department of Urology, Graduate School of Medicine, Chiba University, 1-8-1, Inohana Chuo Ward, Chiba, 260-8677 Japan; 3 External Relations Department, KYOWA HAKKO BIO CO., LTD., 1-6-1, Otemachi, Chiyoda-ku, Tokyo, 100-8185 Japan

As calcium oxalate is the most common constituent of urinary stones animal model of the urolithiasis has been established by feeding l-hydroxyproline, an endogenous precursor of oxalate. However, the influence of oral l-hydroxyproline on human urinary oxalate excretion has not been clearly demonstrated. We herein report a clinical study examining the influence of daily ingestion of l-hydroxyproline on urinary oxalate levels with a focus on measuring oxalic acid excreted in the urine.

In detail, we performed a single-blind, parallel designed study at Sta. Rosa Hospital and Medical Center, Philippines. This study was approved by the hospital review board and was in compliance with the Declaration of Helsinki. Forty healthy subjects (20 males and 20 females, 33.4 ± 9.4 years of age, who has no history of kidney disease) were divided into 4 groups with 10 subjects in each group with same sex ratio. l-Hydroxyproline doses of 0, 500, 1000 and 2000 mg were taken daily after breakfast for 12 weeks. Prior to and during each scheduled examination, subjects were reminded to refrain from intake of oxalate-rich foods and to drink more than 1 L of water per day. Medical interviews, physical examinations, vital sign measurements, anthropometric measurements, blood examinations and 24-h urine sample collections were conducted every 4 weeks at the hospital. A male and two female subjects were withdrawn from this study because of pregnancy or urinary tract infection so the final number of subjects was 37. Informed consent was obtained from qualified individuals. The baseline characteristics of subjects of each group were not different statistically. No adverse events were observed throughout the trial period except for a few symptoms like itchiness or headache which were self-reported from subjects, but the relationships with the test material were denied by the principle investigator.

On the 24-h urine test, almost no markers changed, but there was a significant change in total l-hydroxyproline excretions in the 2000 mg l-hydroxyproline group compared to control group at the point of 8 weeks after first intake. Moreover, oxalic acid levels also increased significantly in the 2000 mg group at the point of 8 and 12 weeks after first intake. However, all values returned to basal values after 4 weeks from the last l-hydroxyproline intake (Table 1). Various dietary factors can change urine oxalate excretion; one is a high-protein diet. Many studies have addressed urinary oxalate excretion after high-protein intake, but conclusions are still uncertain. For example, Knight et al. [1] not only showed significant changes in urinary oxalate with 5 and 10 g of gelatine administered to healthy subjects, but also indicated that the high-protein diet did not influence total daily urinary oxalate excretion [2]. Collagen contains approximately 10 % of l-hydroxyproline, and the daily turnover of collagen in humans is estimated to be 2–3 g/day so the metabolism of l-hydroxyproline will be 240–420 mg [1]. In this study, the urinary oxalate level following 500 or 1000 mg/day l-hydroxyproline ingestion did not change compared with control but 2000 mg/day l-hydroxyproline ingestion increased the urinary oxalate excretion, but the values did not exceed the level of stone forming individuals with a mean oxalate excretion 40 mg/day [2]. As noted, there are various opinions on the association of dietary and urinary oxalate. Recently, it was reported that the Dietary-Approaches to Stop Hypertension (DASH)-style diet is more effective in reducing calcium oxalate supersaturation than a low-oxalate diet, although it includes a lot of fruits and vegetables which contain much oxalate [3]. Thus, the influence of the diet in conjunction with oxalate is still unclear in human metabolism. Moreover, we found no accumulation of l-hydroxyproline and oxalate in the withdrawal period same as the animal model. The metabolic significance of l-hydroxyproline is still unclear and further investigation is warranted.

**Table 1:** Urinalysis results of 24-h urine sample

Urinalysis of 24-h urine	Subjects (*n* = 37: 19 male, 18 female)			
	l-Hydroxyproline			
	0 mg/day (*n* = 10: 5 M, 5 F)	500 mg/day (*n* = 9: 5 M, 4 F)	1000 mg/day (*n* = 10, 5 M, 5 M)	2000 mg/day (*n* = 8: 4 M, 4 F)
Na (g/day)				
Pre-intake	3.1 ± 1	2.8 ± 1.2	3.2 ± 0.9	3 ± 0.8
4 weeks after first intake	3 ± 1	3.5 ± 1.2	3.6 ± 1.1	4 ± 0.6
8 weeks after first intake	2.3 ± 1	1.9 ± 0.8	3 ± 1	2.7 ± 0.7
12 weeks after first intake	3.3 ± 1	3.6 ± 1.2	3.3 ± 0.9	3.9 ± 1.3
4 weeks after final intake	3.5 ± 1.6	3.1 ± 0.7	3.4 ± 0.8	3.3 ± 1.2
K (g/day)				
Pre-intake	0.67 ± 0.27	0.66 ± 0.28	0.82 ± 0.25	0.72 ± 0.24
4 weeks after first intake	0.67 ± 0.2	0.6 ± 0.27	0.7 ± 0.22	0.81 ± 0.25
8 weeks after first intake	0.56 ± 0.19	0.55 ± 0.3	0.56 ± 0.22	0.61 ± 0.18
12 weeks after first intake	0.68 ± 0.29	0.68 ± 0.24	0.61 ± 0.24	0.71 ± 0.28
4 weeks after final intake	0.7 ± 0.3	0.59 ± 0.29	0.67 ± 0.19	0.66 ± 0.3
Ca (g/day)				
Pre-intake	0.16 ± 0.07	0.13 ± 0.06	0.12 ± 0.04	0.11 ± 0.04
4 weeks after first intake	0.19 ± 0.08	0.16 ± 0.04	0.17 ± 0.05	0.17 ± 0.05
8 weeks after first intake	0.12 ± 0.04	0.1 ± 0.03	0.12 ± 0.05	0.12 ± 0.04
12 weeks after first intake	0.14 ± 0.08	0.15 ± 0.08	0.13 ± 0.05	0.14 ± 0.07
4 weeks after final intake	0.13 ± 0.07	0.12 ± 0.09	0.12 ± 0.05	0.13 ± 0.04
Mg (g/day)				
Pre-intake	0.04 ± 0.02	0.05 ± 0.03	0.06 ± 0.02	0.04 ± 0.01
4 weeks after first intake	0.05 ± 0.02	0.05 ± 0.02	0.07 ± 0.02	0.06 ± 0.02
8 weeks after first intake	0.04 ± 0.01	0.05 ± 0.02	0.05 ± 0.01	0.05 ± 0.02
12 weeks after first intake	0.05 ± 0.02	0.06 ± 0.02	0.06 ± 0.01	0.05 ± 0.02
4 weeks after final intake	0.04 ± 0.01	0.05 ± 0.02	0.06 ± 0.01*	0.05 ± 0.02
Inorganic phosphorus (g/day)				
Pre-intake	0.47 ± 0.21	0.41 ± 0.18	0.52 ± 0.12	0.47 ± 0.1
4 weeks after first intake	0.48 ± 0.18	0.45 ± 0.12	0.54 ± 0.08	0.55 ± 0.16
8 weeks after first intake	0.57 ± 0.2	0.49 ± 0.1	0.6 ± 0.22	0.59 ± 0.13
12 weeks after first intake	0.55 ± 0.27	0.51 ± 0.2	0.54 ± 0.13	0.51 ± 0.17
4 weeks after final intake	0.48 ± 0.14	0.41 ± 0.18	0.51 ± 0.16	0.49 ± 0.16
Uric acid (g/day)				
Pre-intake	0.19 ± 0.06	0.15 ± 0.06	0.17 ± 0.08	0.13 ± 0.04
4 weeks after first intake	0.19 ± 0.05	0.17 ± 0.07	0.17 ± 0.06	0.15 ± 0.04
8 weeks after first intake	0.12 ± 0.03	0.09 ± 0.03	0.12 ± 0.06	0.11 ± 0.04
12 weeks after first intake	0.15 ± 0.05	0.13 ± 0.03	0.14 ± 0.04	0.14 ± 0.06
4 weeks after final intake	0.16 ± 0.03	0.11 ± 0.03	0.14 ± 0.05	0.12 ± 0.05
Urea nitrogen (g/day)				
Pre-intake	6.14 ± 2.78	5.9 ± 2.45	6.7 ± 2.02	5.67 ± 1.6
4 weeks after first intake	6.74 ± 2.44	6.29 ± 1.63	7.17 ± 1.37	7.42 ± 2.08
8 weeks after first intake	7.04 ± 2.65	6.53 ± 1.68	7.19 ± 1.9	7.71 ± 1.23
12 weeks after first intake	6.84 ± 2.92	6.78 ± 1.34	6.96 ± 1.58	6.87 ± 2.06
4 weeks after final intake	6.08 ± 1.43	6.15 ± 1.71	6.73 ± 1.24	6.05 ± 2.34
Creatinine (g/day)				
Pre-intake	0.94 ± 0.42	0.82 ± 0.33	1 ± 0.26	0.93 ± 0.28
4 weeks after first intake	1.05 ± 0.37	0.93 ± 0.21	1.14 ± 0.27	1.13 ± 0.33
8 weeks after first intake	1.18 ± 0.46	1.1 ± 0.22	1.28 ± 0.32	1.3 ± 0.24
12 weeks after first intake	1.05 ± 0.47	1.17 ± 0.28	1.15 ± 0.26	1.1 ± 0.44
4 weeks after final intake	1.11 ± 0.34	1.05 ± 0.39	1.18 ± 0.34	1.14 ± 0.45
Albumin (mg/g Cr)				
Pre-intake	2.1 ± 0.4	2.6 ± 1.7	1.9 ± 0.5	1.8 ± 0.5
4 weeks after first intake	2.1 ± 1.1	2 ± 0.4	1.7 ± 0.5	1.8 ± 0.4
8 weeks after first intake	1.9 ± 0.9	1.2 ± 0.3	1.7 ± 0.9	1.5 ± 0.7
12 weeks after first intake	1.9 ± 0.9	1.5 ± 0.3	3 ± 4.5	1.6 ± 0.4
4 weeks after final intake	3.5 ± 4.9	1.6 ± 0.4	1.5 ± 0.5	1.5 ± 0.3
Citric acid (mg/g Cr)				
Pre-intake	248.1 ± 209.2	244.4 ± 156.5	256.3 ± 228.3	212.7 ± 181.2
4 weeks after first intake	244.6 ± 178.7	203.8 ± 137.4	226.2 ± 183.6	175.1 ± 132.4
8 weeks after first intake	197.5 ± 142.2	180 ± 150.7	144.7 ± 114.5	134.9 ± 118.8
12 weeks after first intake	228.7 ± 181.9	189.2 ± 142.2	164.6 ± 140.5	155.5 ± 146
4 weeks after final intake	231.5 ± 176.9	190.6 ± 120.4	214.1 ± 179.6	165.5 ± 118.3
Hydroxyproline (μmol/day)				
Pre-intake	210 ± 89	200 ± 90	232 ± 65	194 ± 51
4 weeks after first intake	218 ± 68	212 ± 48	256 ± 48	288 ± 86
8 weeks after first intake	253 ± 96	244 ± 62	288 ± 72	364 ± 94*
12 weeks after first intake	207 ± 103	241 ± 61	263 ± 93	263 ± 80
4 weeks after final intake	236 ± 88	224 ± 90	246 ± 52	241 ± 74
Oxalic acid (mg/day)				
Pre-intake	15.1 ± 7	12 ± 4.5	15.6 ± 4.1	13.1 ± 2.4
4 weeks after first intake	24.8 ± 7.8	21.5 ± 4.8	27.2 ± 5.6	32.6 ± 8.7
8 weeks after first intake	17.3 ± 4.6	17 ± 4.2	21.5 ± 6.8	24.7 ± 3.7*
12 weeks after first intake	19.2 ± 7.7	23.7 ± 5.9	25.2 ± 5.1	27.1 ± 6.9*
4 weeks after final intake	18.1 ± 6.4	17.5 ± 6.6	18.7 ± 4.7	16.3 ± 6.3

Mean group values and standard deviations were calculated and analyzed for homogeneity of variance using Bartlett’s test (5 % level of significance). When the variance was homogeneous between the groups, mean group values were compared between the control and each treated group using Dunnett’s test. When the variance was heterogeneous based on Bartlett’s test, a mean rank test of the Dunnett type was conducted. Differences from the control group were evaluated at the two-tailed 5 % level of significance and presented as *p < 0.05
